# Protocols for Visually Guided Navigation Assessment of Efficacy of Retina-Directed Cell or Gene Therapy in Canines

**DOI:** 10.3389/fnins.2017.00215

**Published:** 2017-04-26

**Authors:** Puya Aravand, Pavitra S. Ramachandran, Ivan Schpylchak, Nicholas T. Phelps, Sergei Nikonov, Jean Bennett

**Affiliations:** Department of Ophthalmology, Center for Advanced Retinal and Ocular Therapeutics, F.M. Kirby Center for Molecular Ophthalmology, Scheie Eye Institute, University of Pennsylvania Perelman School of MedicinePhiladelphia, PA, USA

**Keywords:** animal model, retinal degeneration, dog, canine, visual function, gene therapy, cell therapy, non-invasive

## Abstract

There has been marked progress in recent years in developing gene delivery approaches for the treatment of inherited blinding diseases. Many of the proof-of-concept studies have utilized rodent models of retinal degeneration. In those models, tests of visual function include a modified water maze swim test, optokinetic nystagmus, and light-dark activity assays. Test paradigms used in rodents can be difficult to replicate in large animals due to their size and awareness of non-visual aspects of the test system. Two types of visual behavior assays have been utilized in canines: an obstacle avoidance course and a forced choice Y maze. Given the progress in developing cell and gene therapies in large animals, such tests will become more and more valuable. This study provides guidelines for carrying out such tests and assesses the challenges and benefits associated with each test.

## Introduction

Studies in canines with a spontaneous mutation in a retina-specific gene encoding retinal pigment epithelium 65 kDa protein (*RPE65*), led the way to the first clinical trials testing gene therapy for treatment of an early onset blindness. Tests of both retinal and visual function were carried out in these animals using electroretinography and navigation through a maze, respectively (Acland et al., 2001; Bennicelli et al., 2008) The results of these and other visual function tests originally carried out in blind dogs served as the basis of proof-of-concept data prerequisite to clinical trials. Improvement in navigational abilities of humans suffering from impaired vision, such as what have been reported for a phase III clinical trial for *RPE65* deficiency, is perceived as an outcome that is clinically meaningful (Maguire et al., 2015; Russell et al., 2016) Assuming that results continue to be positive, this study may lead to the first approved gene therapy product in the USA and the first approved gene therapy for retinal disease in the world. This could serve as an incentive to progress many other preclinical studies to human clinical trials and boost the efforts underway for the ~2 dozen human retinal gene and cell therapy clinical trials in progress^1^.

While many groups have developed the necessary preclinical data carrying out visual function studies in rodents (for example, modified water maze testing, light-dark testing, optokinetic nystagmus) it is very difficult to extrapolate such tests to large animal models due to their larger size and heightened awareness of their surroundings and handlers. Here we describe the basic principles of two test paradigms for navigational vision that can be used in dogs. Both have been or are being used to advance preclinical research to clinical studies (Acland et al., 2001; Komaromy et al., 2010; Beltran et al., 2015).

## Materials and methods

The methods used in these studies should be carried out in accordance with the recommendations of the Guide for the Care and Use of Laboratory Animals (NIH) and the Animal Welfare Act and Animal Welfare Regulations of the USDA. The protocols should also be compliant with the appropriate Institutional Animal Care and Use Committee (IACUC). The University of Pennsylvania IACUC has reviewed and approved the protocols and all experimental procedures were conducted in accordance with the recommendations of that Committee (protocols # 805497, 803871).

### Materials: obstacle avoidance course

**Figure 1:**
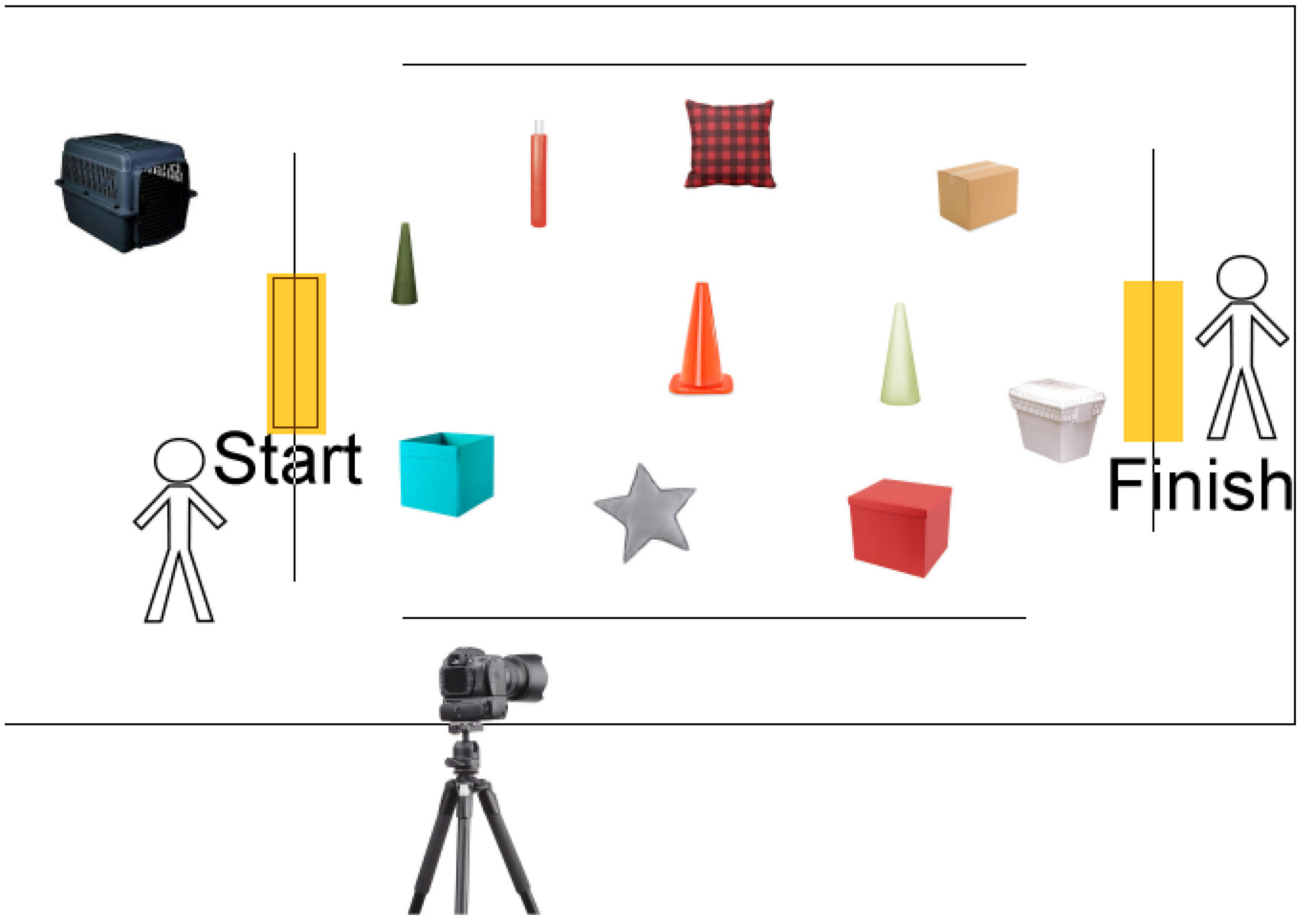
**Prototype for canine obstacle avoidance course**. The 10 washable and non-fragile items are placed within the rectangular framework of the course under dim red lighting by the test givers. This takes place while the animal is dark-adapting in a crate that is draped with an opaque cloth (cloth not shown). After the animal has been dark-adapted and the obstacles have been set up, the animal is led to the “Start” of the course by Tester 1. Tester 2 calls the animal. When the animal reaches the “Finish” point, he/she is given a reward (cuddled or given a treat). An infrared wide-angle camera digitally captures the test results.

A light-tight (windowless) rectangular room of at least 10′ X 15′ is required along with diffuse lighting with distinct set points within a range of 1–4 lux up to 250 lux. The intensities are calibrated and confirmed with a light meter (for example, Extech EA33, FLIR Commercial Systems, Inc., Nashua, NH) at 3 different locations in the room at the level of the animal's head. Ideally the room is insulated to sound from neighboring rooms. The room is equipped with dim red safety lights that the investigators can use to set up the courses while the animal is maintained in a (covered) crate placed at one end of this room. A total of 10 different obstacles that are soft and/or do not cause injury if the animal collides with them are used (for example, a plastic-covered foam cushion, a cardboard box, a traffic cone; Figure 1). The objects should be washable to be able to remove scent from an animal that was tested previously. The setup of the course is randomized from test to test. A digital single lens reflex camera capable of recording HD video is used such that both ends of the course can be visualized simultaneously. Alternatively, an infrared camera (for example, Flir One, Nashua NH) can be used by an observer to record the animal's movements. Each eye can be tested separately by placing a black protective plastic ocular shield (Ambler Surgical, Exton, PA) after topical application of anesthetic [proparacaine hydrochloride ophthalmic (Alcon) or tetracaine hydrochloride (Bausch and Lomb)], and lubricant (for example, Goniosol, hydroxypropyl methylcellulose solution, Alcon, Fort Worth, TX) on the dog's contralateral eye.

### Materials: forced choice Y maze

**Figure 2:**
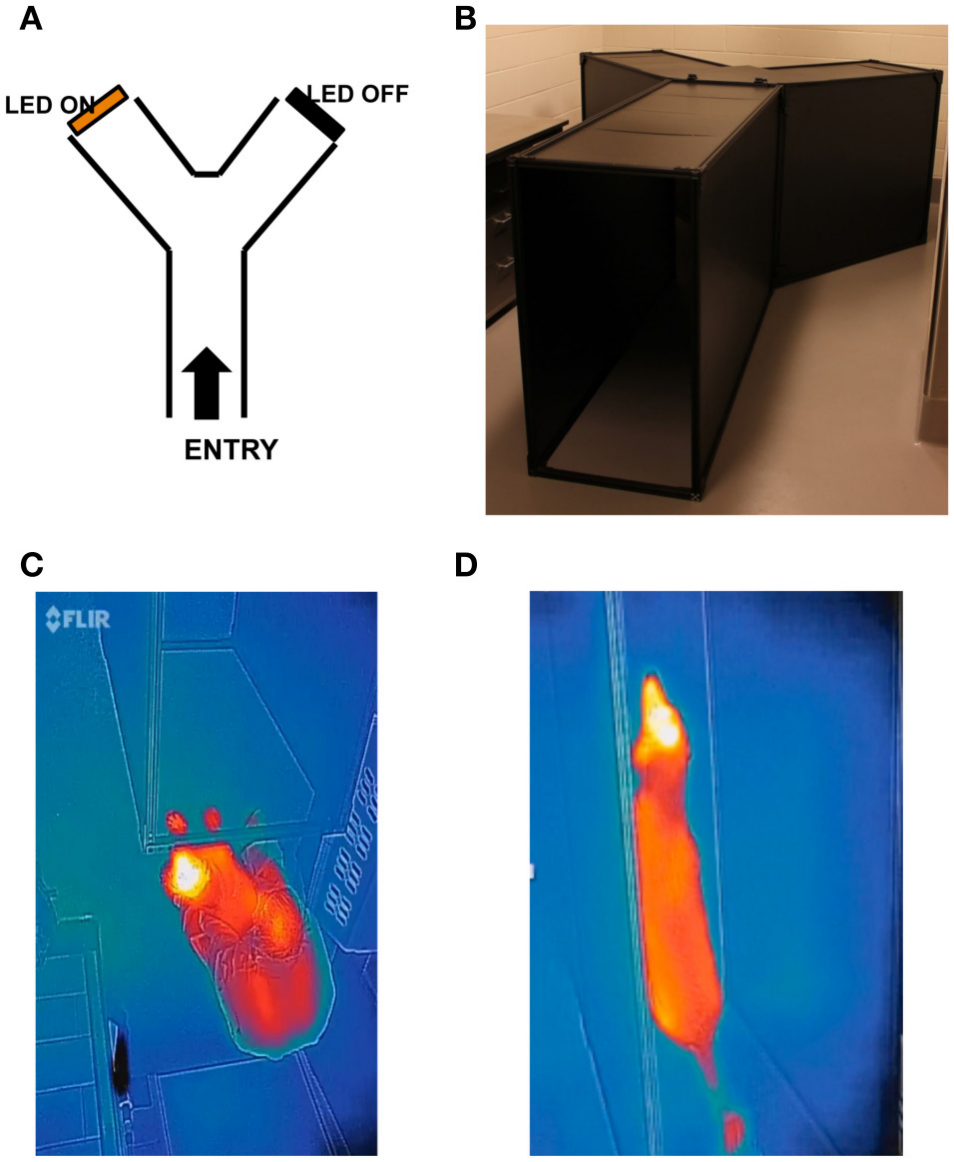
**Prototype for canine forced choice Y maze. (A)** Diagram of Y maze configuration with examples of non-hazardous obstacles; **(B)** Set up of the Y maze; **(C)** Infrared view of animal entering the Y maze; **(D)** Infrared view of the same animal transiting the Y maze. The testers (not shown) are located at the entrance to the maze and on the distal side of the maze at the bifurcation position. The infrared camera (which digitally captures the animal's progress) is not shown.

A maze in the shape of a “Y” is constructed such that the animals traverse a 1.78 m entry path before deciding to turn left or right at the split in the “Y.” The angle between the arms of the Y is ~ 90° so that the angle between the arms and the Y-maze axis is 45°. The height of the maze is 1 m. The distance of each arm of the “Y” is 1.5 m (Figure 2). The Y maze is placed in a light-tight room, and the color of the entire maze is black. The maximum light intensity at the animal's decision point is 0.3 mW/cm^2^, although testing can be run at much lower intensities. Two identical light sources hang from the roof of the maze at the ends of either exit arm.

The frame of the maze is composed of 1″ × 1″ (80/20) aluminum profiles and the sides and walls are removable black plastic panels. Black rubber stripping is placed on the seams to assure that the inner aspect of the maze was isolated from external light. The top is removable and the exits can be obscured with black curtains. Horizontal custom-made strips of tightly packed LEDs (50 LEDs in a strip, 4 mm between LED centers, LED lens diameter around 2.5 mm, 470 nm LUXEON Rebel LXML-PB01-0040 or 590 nm LXM2-PL01-0000 obtained from DigiKey, Thief River Falls, MN) are used to emit light of the appropriate wavelength. (DigiKey, Thief River Falls, MN) are used to emit light of the appropriate wavelength. Light sources (LUXEON Rebel 470 nm LXML-PB01-0040 or 590 nm LXM2-PL01-0000 LEDs) can be used in the Y-maze light stimulation system. The diameter of LED lens should be around 2.5 mm. To insure proper heat dissipation a solid copper plate connected to aluminum case is used as a common heat sink. A heat transfer paste is used between copper plate and heat sinks of individual LEDs. LED strips are driven by a Keithley 2260B-30-36 (or equivalent) programmable power supply and the LED lights and the power supply emit negligible heat under standard light stimulation conditions (Figures 2C,D). The intensities are measured with a calibrated photodiode (OSI Optoelectronics, Hawthorne, CA) at different driving currents, distances and angles from the LED axis. Intensities are confirmed at the start of each individual experiment run with a light meter (for example, Extech EA33, FLIR Commercial Systems, Inc., Nashua, NH).

Details of variations in light intensity are as follows: According to angular measurements, variations in light intensity due to the differences in vertical position of the dog head (assuming the dog is looking at the light source) should be less than 2-fold (in a plane perpendicular to the LED strip, the light intensity declined ~2 × for a ~30 degrees angle). As expected with increased distance *R* between the photodiode and light source, the irradiance declines as ~*R*^−2^. The brightest attainable corneal irradiance at 50″ is 0.35 and 0.39 mW/cm^2^ (for 470 and 590 nm, respectively). The corneal irradiance used in most of the behavioral tests is in the 0.04–0.08 mW/cm^2^ range for both 470 and 590 nm light sources (measured at 50″ from the light source).

The retinal irradiance *E*_*retinal*_ can be estimated from the measured corneal irradiance *E*_*corneal*_ as

$$\begin{array}{l} E_{retinal}=E_{corneal}\times A_{pupil}/A_{image} \end{array}$$

or

$$\begin{array}{l} E_{retinal}=E_{corneal}\times A_{pupil}/\left(50\times A_{image,LED}\right)\\ =1/50\times E_{corneal}\times {\left(D_{pupil}/D_{image,LED}\right)}^{2} \end{array}$$

where *A*_*pupil*_ and *D*_*pupil*_ are the area and diameter of the pupil, *A*_*image*_ is the area of the image of the light source on the retina, and *A*_*image, LED*_ and *D*_*image, LED*_ are the area and diameter of the retinal image of an individual LED.

The diameter of the retinal LED image can be estimated using rules of the geometric optics with further corrections to account for the dog eye point spread function. Due to the similarity in geometry of human and dog eyes, the point spread function of a dog eye is expected to be similar to that of a human eye which ranges from around 0.007 (for pupil diameter ~3–4 mm) to 0.020–0.025 mm for a fully constricted or dilated pupil (Gross et al., 2008). Assuming the focal distance of a dog eye to be around 20 mm, at 50″ (1,270 mm) from the light source (around the point where dog is expected to choose one of the exit channels) the 2.5 mm LED should be focused on the retina to a spot of 2.5 × 20/1270 ≈ 0.04 mm in diameter. Accounting for the point spread function of the constricted pupil will increase the spot diameter to around 0.05–0.06 μm. Thus retinal irradiance for the constricted pupil (*D*_*pupil*_ ~1 mm) can be estimated as *E*_*retinal*_ ≈ 1/50 × E_*corneal*_ × (1/0.06)^2^ ≈ 6 × E_*corneal*._ This translates into maximum retinal irradiance of around 2.1 and 2.3 mW/cm^2^ for 470 and 590 nm light stimuli. The most commonly used range of retinal irradiances can be estimated as 0.24–0.48 mW/cm^2^ or from 5.7e6 to 11.4e6 and from 7.1e6 to 14.2e6 photons s^−1^ μm^−2^ for 470 and 590 nm light, respectively.

At the distances from the light source larger than the light source dimension but short enough for the LED retinal image to be larger than the point spread function of the eye lens, an increase in the distance *R* from the light source to the eye will make corneal irradiance decline as *R*^−^^2^ and at the same time decrease the light source retinal image area as *R*^−^^2^, thus increasing light density on the retina as *R*^2^. As a consequence retinal irradiance will be nearly independent of the distance between the eye and the light source. The above conditions are approximately valid for the area where the dog selects the exit channel. Around that area, variation in the distance from the light source should have little effect on the retinal irradiance.

The light stimulus duration can also be controlled (constant vs. flickering light) if desired. The strips of LEDs are anchored at a height compatible with the dog's eyes and oriented facing the bifurcation in the maze. Each eye can be tested separately by placing a black protective plastic ocular shield (Ambler Surgical, Exton, PA) after topical application of anesthetic [proparacaine hydrochloride ophthalmic (Alcon) or tetracaine hydrochloride (Bausch and Lomb)], and lubricant (for example, hydroxypropyl methylcellulose solution) on the dog's contralateral eye. An infrared camera (for example, Flir One, Nashua NH or Sony Handycam) is used to record the animal's movements.

### Methods: obstacle avoidance course

Animals undergo a minimum of 3 days of training prior to carrying out scored tests. The same two-three personnel give the training and test administration. During this time period, the animals become accustomed to the conditions of the test (placement in a crate, application of the ocular shield), the room, the obstacles, and the test givers. To train for the test, simulations of the course are carried out under high mesopic (250 lux) conditions. Tester A removes the animal from the crate and guides the animal to the “beginning” of the course. Tester B calls the animal from the other side of the room. The obstacles should be placed at designated locations in the path between tester A and tester B. The obstacles should be placed such that the animal can navigate without touching/moving them. The animal receives a treat (hugs, praise, and/or edible treat depending on what seems to gratify the dog) after traversing the course. The process is repeated for each eye.

Once the animal is comfortable with the paradigm, he/she is dark adapted for 20 min and then given the formal tests, starting with eye #1 (chosen randomly). The contralateral eye receives the ocular shield. The test givers are masked as to the intervention status. Each eye is tested three times under 3 distinct ambient illuminations going from the lowest (scotopic) to the highest (mesopic) light levels. The test is administered similarly as the training program with Tester A placing the animal at the “beginning” of the course and the Tester B calling the animal at the “end” of the course. A third person (Tester C) carries out the filming, helps clean/replace dirty obstacles and place the obstacles for each different test run. The process is repeated for the contralateral eye. The room and obstacles are cleaned with an animal friendly disinfectant (a compound that has already been approved by the animal facility) in order to remove distracting smells prior to initiating tests on another animal.

An experienced observer who is masked to the experimental design scores test results. The observer measures both speed and accuracy of the animal navigating the test for each trial. The time that it takes to navigate the test is defined as that between placement of the animal at the entrance of the course by Tester A and the moment that the animal contacts Tester B. Accuracy is defined as the number of collisions with obstacles during transit through the course. The mean difference in speed and accuracy between intervention eyes and contralateral control eyes is calculated and statistical analyses are performed. Five to 10 tests per eye per animal can be performed to calculate statistical significance.

#### Example of results using obstacle avoidance course

Results of visual behavior studies for one animal pre- and post-bilateral gene therapy are provided here. The animal was 4 months old at baseline and homozygous for a four nucleotide (AAGA) deletion (nucleotides 487–490) in the *RPE65* gene. At baseline, the animal was unable to carry out the test under scotopic (5 Lux) or low mesopic (100 Lux) lighting conditions. Under those lighting conditions, the animal lay down and refused to move. Under bright light (250 Lux) conditions and using both eyes (i.e., neither eye patched), the animal completed the course in an average of 9.9 s with an average of 10.45 collisions/test. The animal then received bilateral gene transfer of 1.5E11 vg/eye subretinal AAV2.hRPE65v2 (Bennicelli et al., 2008), a recombinant AAV that delivers the wildtype *hRPE65* cDNA driven by a constitutive (chicken β actin promoter with CMV enhancer) (Amado et al., 2010). Repeat obstacle avoidance course testing was carried out 1 month after gene transfer to the second eye. With an illuminance of 250 Lux, there was an average of 0.5 collisions/test and an average course completion time of 4 s. The difference in number of collisions/test before and after gene therapy was significant (*P* < 0.0001) as was the time required to complete the test (*P* < 0.0001). Further, whereas the animal had been unable to navigate the course under dim light (5 lux) conditions prior to gene therapy, it was able to navigate the course with high accuracy and speed (0 collisions/test and average course completion of 4.0 s) after treatment. In summary, after gene therapy, the animal was much more active under both scotopic and mesopic conditions and showed significant improvement in speed and accuracy in navigating the course compared to baseline.

### Methods: Y maze

The training process prior to carrying out formal Y-maze testing takes a minimum of 5 days, depending on the animal's temperament, and is carried out in stages by the same two testers (Tester A and Tester B). Unilateral ocular shields can be used at each stage of training although this is not advisable if animals have such limited vision that reducing it further renders them fearful. Stage 1 aims to make the animal comfortable entering and traversing the open (and brightly lit) maze. Stage 2 aims to make the animal comfortable entering and traversing the closed maze with room lights on. Stage 3 aims to make the animal comfortable entering and traversing the closed maze with room lights off.

In Stage 1 of training, the roof of the maze is removed and the process is carried out in a brightly lit room (~250 lux ambient light). A random number generator is used to determine which light is used for each test (either “arm 1” or “arm 2”), with an equal number of 1's and 2's used during testing. Tester A places the animal at the maze entrance and Tester B calls the animal from the end of arm of the maze that contains the bright light source. Each time the animal traverses the maze and exits at the end with the light source, they receive treats (affection, praise, and/or edible treats). If they exit at the end of the maze lacking the light source, they do not receive praise/treats. Stage 1 of training typically takes 3–5 days and is complete when the dog is willing to enter and exit the maze independently and is accustomed to placement in the crate, application of the ocular shield (if tolerated, see above), the room, and the test givers. Training is done using each eye individually.

In Stage 2 of training, the same procedures are carried out as Stage 1, except that the roof of the maze is in place. If the animal does not experience placement of the ocular shield in Stage 1 training, that is introduced at this stage. Stage 2 of training typically takes 1–2 days.

In Stage 3 of training, the same procedures are carried out as in Stage 2, except that the room lights are off and the only light is that from the light source placed at the end of one arm of the maze. Stage 3 of training typically takes 1–2 days. By Stage 3 of training, Tester B usually no longer needs to call out for the dog to traverse the maze as the dogs should be comfortable doing this on their own. Tester B from this point on should be at the middle of the 2 arms (Figures 2A,B) and not stand at either end of the arms—which could bias the dog toward the correct exit.

Training is considered complete when the dog is comfortable with all of the procedures and is willing to enter and exit the maze in a darkened room.

In summary, animals undergo a minimum of 3–5 days of training prior to carrying out scored tests. During this time period, they become accustomed to the conditions of the test (placement in a crate, application of the ocular shield to each eye), the room, the obstacles, and the test givers under bright light (250 lux) conditions.

For the formal testing, one eye (randomly selected) is tested at a time. Testing is carried out under zero ambient light conditions, with the only light being that from the light source placed at one arm of the Y maze. A minimum of 20 trials is carried out per eye/light condition. A random number generator is used to determine whether the light is switched on at the “arm 1” vs. “arm 2” exit of the Y maze, with an equal number of 1's and 2's used during testing. The process is then repeated for the contralateral eye. Repeat testing is performed under the subsequent set of light conditions, again using 10–20 trials per condition/eye. Typically, the progression of testing conditions goes from low to bright lighting. The room and Y-maze are cleaned with disinfectant in order to remove distracting smells prior to initiating tests on another animal.

An experienced observer who is masked to the experimental design scores test results. The observer measures both speed and accuracy of the animal in navigating the test for each trial. The time that it takes to navigate the test is defined as that between placement of the animal at the entrance of the course and the moment that the animal completely passes through the exit. Accuracy is defined as making the correct choice to the arm of the maze that contains the light stimulus (i.e., the proportion of correct exit choices). The mean difference in speed and accuracy between intervention eyes and contralateral control eyes is calculated and statistical analyses are performed.

## Discussion

With recent progress in development of cell and gene therapies, there is more and more interest in testing interventions that could be used to treat both specific genetic diseases affecting vision or to resuscitate visual pathways after photoreceptors have degenerated. Large animal models are attractive targets for such studies for many reasons, including the facts that they have anatomical features similar to those of humans, similar surgical procedures can be used to deliver the intervention in large animal models as would be used in humans, they have similar immunologic responses to those experienced by humans, and unlike rodents, they are light seeking (instead of light avoiding).

While one might expect that it is easy to develop tests of visual behavior in large animal models, there are certain challenges imposed by the size of the animals and their interactions with their surroundings and handlers. In initial studies, we described observational studies of navigation of a canine model of a severe early onset retinal dystrophy before and after gene augmentation therapy (Acland et al., 2001). Komaromy et al. and Banin et al. modified this test for application under bright light conditions in order to demonstrate efficacy after gene augmentation therapy for congenital *CNGB3*-associated achromatopsia in dogs and sheep, respectively (Komaromy et al., 2010; Banin et al., 2015). In the process, Komaromy and colleagues rendered the test more quantitative (Garcia et al., 2010). The modified test paradigm is an obstacle avoidance course that is 3.6 m in length. The obstacles consist of 6 moveable white polyvinyl chloride panels placed on steel shelving tracts. These panels do not completely obscure the path, but leave a 0.3 m space through which the animal can continue to ambulate forward. The panels can be moved from side to side, thereby allowing the dog to navigate through the respective “doors” created by the moved panels. Garcia et al. showed that the achromatopsia dogs have difficulty navigating around the obstacle panels at the higher light intensities.

The moveable panel test has since been used to gather data assessing navigational abilities in canine models of X-linked and autosomal dominant retinitis pigmentosa (Beltran et al., 2015; Iwabe et al., 2016). It serves as a potential alternative to the “random object” mobility course. Potential advantages of using the “random object” obstacle course over the moveable panel test are that the course can be set up (and dismantled) easily, it is inexpensive, it does not require extensive training, and it is not affected by the possibility that the animals navigate through echolocation (i.e., using auditory clues) or by feeling the panels with their paws. The course still presents challenges, however, as some animals are inherently more timid or more easily distracted, and those qualities can affect the outcome of the test. The same temperamental effects would likely be true for the moveable panel test.

Studies using a forced choice (Y maze) navigational test in canines have also been reported. The Y maze has been used in canine studies of gene augmentation therapy and in optogenetic therapy (Beltran et al., 2015). The results reported to date show an improved ability to detect light stimuli in eyes of XLRP dogs treated with gene augmentation therapy. It should be noted for this forced choice test that the animals have a 50:50 chance of making the correct choice even without training, so that it is important to carry out a sufficient number of tests to demonstrate any statistically significant improvement over chance. Another potential disadvantage of the forced choice test is that it requires substantially more training effort than the other tests.

In summary, here we describe the set-up, training plan and potential challenges of different tests of visual behavior that can be used in canines maintained in the laboratory. The focus of these tests is the ability to navigate accurately and confidently using visual cues—a skill that is meaningful to both animals and humans. The detailed protocols described here are meant to serve as a starting point for others to establish navigational tests relevant to different large animal models. Modifications may be required for individual animals (due to differences in temperament) or to adapt the protocol to test for presence (or correction) of the relevant visual deficits. The test paradigms take advantage of the social interactions most canines have with humans and minimize the possibility that test outcomes could be affected by their other senses (smell, hearing) and interactions with other dogs. The test paradigms are non-invasive, fun for the animals, and require only a large light-tight room and minimal supplies. The surfaces of the testing devices can be cleaned to minimize the possibility that other senses are assisting the dog to navigate correctly (or even potentially distracting the animal!). Further, the entire training and test administration process can be completed for most animals in 1 week's time. We believe that the experience that we have accumulated working with these precious animals will be useful to others for advancing preclinical research to clinical studies. The tests could also be adapted for other large animal species. These tests will provide the critical preclinical data indicating whether it is worthwhile to proceed to human clinical trials for particular modalities.

## Author contributions

PA, PR, IS, NP, SN, and JB participated in the design of the assays and experimental set-up. PA, PR, IS, NP, and JB acquired data and PA, PR, and JB analyzed data. JB obtained regulatory approvals. JB wrote the manuscript and all other authors reviewed and edited the intermediate and final version.

### Conflict of interest statement

JB is a founder of Gensight Biologics and of Limelight Bio and a scientific (non-equity-holding) founder of Spark Therapeutics. The other authors declare that the research was conducted in the absence of any commercial or financial relationships that could be construed as a potential conflict of interest.

## Footnotes

^1^www.clinicaltrials.gov.
